# Immunization with a Neural-Derived Peptide Protects the Spinal Cord from Apoptosis after Traumatic Injury

**DOI:** 10.1155/2013/827517

**Published:** 2013-10-23

**Authors:** Roxana Rodríguez-Barrera, Ana M. Fernández-Presas, Elisa García, Adrian Flores-Romero, Susana Martiñón, Viridiana Yazmín González-Puertos, Humberto Mestre, Carmina Flores-Dominguez, Verónica Rodriguez-Mata, Mina Königsberg, Sandra Solano, Antonio Ibarra

**Affiliations:** ^1^Facultad de Ciencias de la Salud, Universidad Anáhuac México Norte, Edo. de México, CP 52786, Mexico; ^2^Departamento de Ciencias de la Salud, División de Ciencias Biológicas y de la Salud, Universidad Autónoma Metropolitana Iztapalapa, DF, CP 09340, Mexico; ^3^Centro de Investigación del Proyecto CAMINA A.C., DF, CP 14050, Mexico; ^4^Posgrado en Biología Experimental, Universidad Autónoma Metropolitana Iztapalapa, DF, CP 09340, Mexico; ^5^Departamento de Microbiología y Parasitología, Facultad de Medicina, UNAM, DF, CP 04510, Mexico

## Abstract

Apoptosis is one of the most destructive mechanisms that develop after spinal cord (SC) injury. Immunization with neural-derived peptides (INDPs) such as A91 has shown to reduce the deleterious proinflammatory response and the amount of harmful compounds produced after SC injury. With the notion that the aforementioned elements are apoptotic inducers, we hypothesized that INDPs would reduce apoptosis after SC injury. In order to test this assumption, adult rats were subjected to SC contusion and immunized either with A91 or phosphate buffered saline (PBS; control group). Seven days after injury, animals were euthanized to evaluate the number of apoptotic cells at the injury site. Apoptosis was evaluated using DAPI and TUNEL techniques; caspase-3 activity was also evaluated. To further elucidate the mechanisms through which A91 exerts this antiapoptotic effects we quantified tumor necrosis factor-alpha (TNF-**α**). To also demonstrate that the decrease in apoptotic cells correlated with a functional improvement, locomotor recovery was evaluated. Immunization with A91 significantly reduced the number of apoptotic cells and decreased caspase-3 activity and TNF-**α** concentration. Immunization with A91 also improved the functional recovery of injured rats. The present study shows the beneficial effect of INDPs on preventing apoptosis and provides more evidence on the neuroprotective mechanisms exerted by this strategy.

## 1. Introduction

Apoptotic cell death is one of the main destructive phenomena triggered after brain and spinal cord (SC) injury [1, 2]. This phenomenon is known to be activated by inflammatory cytokines, free radicals, excitotoxic agents, and increased levels of intracellular calcium [3]. All of these factors are seen after SC injury. Apoptosis is characterized by chromatin fragmentation, condensation, and appearance of apoptotic bodies seen as a small basophilic material within the nucleus or as basophilic material extruded from the cell within cytoplasm blebs [4]. As early as 4 hours after SC injury, apoptotic cells are observed at the injury site, and they continue to appear in a time-dependent manner. Their distribution follows a centrifugal pattern from the epicenter in both rostral and caudal directions [3, 5].

After SC injury, neurological recovery depends mainly on the extent of neuronal loss and the functionality of the residual neural tissue. Numerous studies showed that many neurons die as a consequence of apoptosis. Therefore, regulating apoptotic cell death might play an important role in the neurological recovery following SC injury [6, 7].

Recent studies have suggested that modulation, rather than suppression, of immune response could be the best way to attain neuroprotection and neuroregeneration after SC injury [8–10]. Research in this field has shown that immunization with neural-derived peptides (INDPs) could provide the necessary conditions to achieve the beneficial and avoid the detrimental effect of immune cells. Vaccination with A91, a nonencephalitogenic myelin-basic-protein (MBP) derived peptide, has shown to improve motor recovery and reduce tissue damage after SC contusion [11]. Furthermore, the therapeutic window of this strategy allows its combination with other therapies without avoiding its beneficial actions [12]. In some cases, it has even demonstrated synergistic properties resulting in an improved functional outcome [11].

The mechanism by which A91 achieves its beneficial effects has been the aim of recent studies. For instance, it has been shown that A91 diminishes lipid peroxidation [8]. This effect is due in part to a reduction in nitric oxide (NO) production and inducible nitric oxide synthase (iNOS) gene expression [13]. The immune response elicited by A91 displays a Th2 phenotype, that is, capable of releasing brain-derived neurotrophic factor (BDNF) [14].

Searching to shed light on other neuroprotective effects exerted by A91, we studied the effect of immunization on apoptosis after SC injury. The basis of this investigation relies on the fact that free radicals, especially NO, trigger forms of programmed cell death, such as apoptosis [15, 16]. Upon seeing that A91 reduces NO [12] and increases the release of BDNF, an antiapoptotic molecule [14, 17], we hypothesized that immunizing with this peptide might be capable of reducing apoptosis triggered by SC injury. To further elucidate the specific mechanisms through which A91 exerts this antiapoptotic effect, tumor necrosis factor-alpha (TNF-*α*) was quantified. TNF-*α* is a byproduct of the proinflammatory response and a ligand of TNF receptor 1 (TNFR1), a member of the death receptor family [18]. Increased NO and iNOS expression after SCI is responsible for TNF-*α*-mediated apoptosis [19]. A91 has demonstrated to lower NO and iNOS and deviate the immune response towards an anti-inflammatory phenotype; all these factors may correlate with a decrease in total TNF-*α* concentrations and therefore result in less TNF-*α*-mediated apoptosis.

## 2. Materials and Methods

### 2.1. Experimental Animals

The Animal Breeding Center of Camina Research Project supplied us with adult female Sprague-Dawley (SPD) rats (13-14 weeks old, 200–220 g). The rats were age-matched and housed in a light- and temperature-controlled room. Efforts were made to minimize the number of animals used, as well as their suffering. All procedures were in accordance with the National Institutes of Health (US) Guide for the Care and Use of Laboratory Animals and the Mexican Official Norm on Principles of Laboratory Animal Care.

### 2.2. Study Design

Three independent sets of experiments were performed. For the first one, 36 animals were allocated into three groups (*n* = 12 per group): (1) spinal cord injury plus immunization with A91; (2) spinal cord injury plus immunization with vehicle only; and (3) sham-operated rats that received no immunization. Seven days after the surgical procedure, animals were euthanized for morphological (DAPI stain; *n* = 6 per group) and immunochemical (Western blot assay; *n* = 6 per group) studies. For the second experiment, 12 rats were allocated into 3 groups (*n* = 4 per group) as was displayed before. Seven days after surgical procedure, animals were euthanized to evaluate apoptosis by the TUNEL assay. Finally, in the third experiment, 24 rats were subjected to SC contusion and then allocated into two groups (*n* = 12 per group): (1) spinal cord injury plus immunization with A91 and (2) spinal cord injury plus immunization with vehicle. Thirty days after SC injury, both groups were evaluated for motor recovery.

### 2.3. Spinal Cord Injury

Rats were subjected to SC contusion as previously described [8]. Thirty minutes after an intramuscular injection of ketamine (50 mg/kg, Probiomed, Mexico City, DF, Mexico) and xylazine (10 mg/kg; Fort Dodge Laboratories, Fort Dodge, IA, USA), a 10 g. rod was dropped onto the spinal cord from a height of 25 mm using the NYU impactor (NYU, New York, NY, USA). This device has shown to inflict a well-calibrated contusive injury of the SC. Surgical access to the spinal cord was achieved with a laminectomy of the T9 vertebral body; the contusive injury was inflicted at this level as well.

### 2.4. Immunizations

Immediately after injury (no longer than 60 min after injury), animals were injected subcutaneously at the base of the tail with either 150 *μ*g of A91 (Invitrogen Life Technologies, San Diego, CA, USA) or phosphate buffered 0.15 M saline, pH 7.4 (PBS) as a vehicle. All immunizations were emulsified in an equal volume of complete Freund's adjuvant (CFA, Sigma, St. Louis, MO, USA) containing 0.5 mg/mL *Mycobacterium tuberculosis.*

### 2.5. Morphological and Immunochemical Evaluations

Seven days after sham operation or SC contusion, animals were anesthetized, and two centimeters of fresh SC from the site of injury were dissected from each rat for morphological and immunochemical studies. After the samples were obtained, the animals were euthanized, and the tissue was stored at −70°C until its use.

### 2.6. DAPI Stain

The SC was processed for DAPI staining according to the method previously described [20]. SC was frozen at −70°C and embedded in tissue freezing medium (Tissue Tek). Three serial 6 *μ*m thick sections were obtained from the epicenter and another three 2 mm away from the epicenter in a caudal direction and another three 2 mm rostrally. Sections were prepared in a cryostat and deposited on glass slides covered with poly-L-lysine (Sigma Chemical Co, St. Louis, MO, USA) and kept at −70°C until use.

Frozen sections of the SC were fixed using 3.7% formaldehyde in PBS (15 min 23°C) followed by incubation in 0.1% Triton X-100 to permeabilize the nuclei. In order to observe the nuclear morphology of cells, frozen sections were exposed for 15 min at 37°C to selectively histochemical labeling with 0.1 *μ*g/mL 4′,6 diamidino-2′-phenylindole diHCL, a fluorescent dye that selectively labels DNA (DAPI, Boehringer, Indianapolis, IN, USA); sections were counterstained with Evans Blue. Finally, after washing with PBS pH 7.4, sections were air-dried and mounted with VECTASHIELD mounting medium (Vector, Burlingame, CA, USA) and analyzed by a blinded observer in an epifluorescence microscope. Sixty random fields (twenty fields per section) at 60x from each experimental and control specimen were analyzed. The final average of apoptotic cells from each analyzed level (epicenter and 2 mm caudal-rostral to the epicenter) was obtained from the total number of cells observed in each field. Apoptotic cells were identified by condensation and fragmentation of the nuclei (separated nuclei composed by clusters of blue dots).

### 2.7. Deoxynucleotidyl Transferase-Mediated Nick-End Labeling (*TUNEL*) Technique

DNA fragmentation was analyzed using the terminal-deoxynucleotidyl-transferase-(TdT-) mediated nick-end labeling technique, TUNEL (Boehringer Mannheim, Indianapolis). Frozen sections were covered in freshly prepared 4% paraformaldehyde solution in PBS, pH 7.4 and incubated for 25 min at room temperature in a slow-moving shaker and washed three times for 10 min in PBS. Slides were covered with permeabilization solution (0.1% Triton X-100 in 0.1% sodium citrate) for 9 min at room temperature, washed twice with PBS, covered with 50 *μ*L of TUNEL reaction mixture, and incubated for 60 min at 37°C in a humidified light-proof box. Samples were washed twice in PBS, covered with a 10% solution of Evans Blue for 10 min (to block autofluorescence), and rinsed once. Sections were analyzed in an epifluorescent microscope, using an excitation wavelength in the range of 450–500 nm and detection in the range of 515–565 nm. As for the negative controls, fixed and permeabilized slides were incubated in 50 *μ*L of label solution without TdT. To induce DNA strand breaks for the positive control, the fixed and permeabilized slides were exposed to DNAse I, grade I (Sigma) (3000 U/mL in 50 mM Tris-HCl; pH 7.5; 1 mg/mL BSA) for 30 min at room temperature. Sixty random fields (twenty fields per section) at 60x from each experimental and control specimen were analyzed. Images were analyzed with the ImageJ 1.47 Program.

### 2.8. Protein Extraction and Western Blot Analysis

Proteins were extracted from SC sections of sham and experimental animals (*n* = 6 per group) using a T-PER Extraction Kit (no. 78510, Thermo Fischer Scientific) with complete protease inhibitor (Santa Cruz Biotechnology, Inc.). Total protein concentration was determined spectrophotometrically at 595 nm using a commercial Bradford reagent (Bio-Rad, Hercules, CA, USA) (Bradford, 1976). Caspase-3 and TNF-*α* were quantified using Western Blot and specific antibodies as described elsewhere [21]. Proteins were separated on 12% SDS-PAGE gels [22], transferred to polyvinylidene difluoride (PVDF) membranes (Invitrogen), and probed with anti-caspase-3 and anti-TNF-*α* antibodies (Santa Cruz Biotechnology, Santa Cruz, CA, USA). Equal loading was demonstrated by probing the same membranes with an antiactin antibody as a housekeeping protein (donated by Dr. A. Hernández, Cinvestav, IPN, Mexico). Membranes were washed three times with TBS-Tween and incubated with anti-mouse IgG secondary antibody (Pierce Biotechnology, Rockford, IL, USA) for 1 h. After three consecutive washes, the blots were developed using a commercial chemiluminescence reagent (SuperSignal West Pico Chemiluminescent Substrate, Thermo Fischer Scientific). The proportion of these proteins was quantified by densitometric analysis, using Kodak IMAGEN GEL DOC and its respective software (v. 3.1).

### 2.9. Assessment of Motor Recovery

Behavioral recovery was assessed using the Basso, Beattie, and Bresnahan (BBB) open field test of locomotor ability [23]. Recovery was scored on the BBB locomotor rating scale of 0 (complete paralysis) to 21 (complete mobility). Observers blinded to the treatment received by each rat performed the test. Evaluations were held 30 days after SC injury in order to correlate a reduction in apoptosis with functional recovery. For a more detailed evaluation of A91-induced neurological recovery please refer to [8, 13, 14].

## 3. Results

### 3.1. A91 Immunization Reduced Apoptotic Cells at the Site of Injury

**Figure 1:**
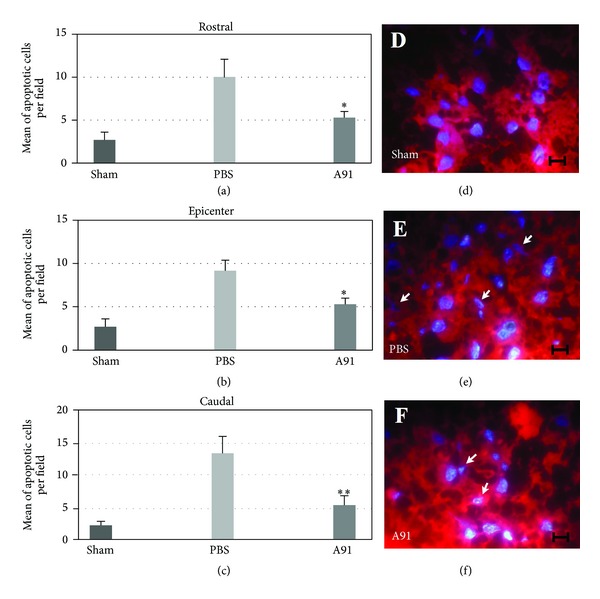
Number of apoptotic cells at the site of injury. Seven days after SC injury. (a) Rostral region 2 mm from injury epicenter. (b) Injury epicenter at T9 spinal cord level. (c) Caudal region 2 mm from injury epicenter. A91 immunization induced a significant reduction in apoptotic cells. *Different from PBS-immunized rats (*P* < 0.001; Student's *t*-test); **Different from PBS-immunized rats (*P* < 0.0001; Student's *t*-test). Bars represent the mean ± SD of 6 rats. DAPI stain micrograph depicting apoptosis as chromatin condensation and fragmentation of the injury epicenter at 7 days after SC injury. (d) Sham-operated animals had an average of 2.7 ± 1 apoptotic cells at the injury site. (e) Animals subjected to SC contusion and posterior immunization with PBS demonstrated an average of 9.17 ± 1.2 apoptotic cells at the epicenter. (f) The experimental group consisting of A91 immunization had 5.3 ± 0.7 apoptotic cells per field. Arrows depict examples of cells undergoing apoptosis identified by chromatin fragmentation, condensation, and appearance of apoptotic bodies seen as small DAPI-stained clusters within the nucleus or extruded from the cell within cytoplasm blebs. Scale bar = 10 *μ*m.

Figure 1 shows the number of apoptotic cells using the DAPI technique. This depicts that A91 immunization induced a significant reduction of apoptotic cells both at the epicenter and at 2 mm rostral and caudal to this site. In the epicenter, the average of apoptotic cells in sham-operated rats was 2.7 ± 1 (mean ± standard deviation (SD); number of cells per field; see Figure 1(d)), while in the case of SC-injured animals immunized with PBS, the average was 9.17 ± 1.2 (Figure 1(e)). A91 immunization reduced the amount of apoptotic cells to 5.3 ± 0.7 (*P* = 0.001  *versus* PBS; Student's *t*-test; Figure 1(f)). In the case of 2 mm rostral to the epicenter, the average of apoptotic cells was sham 2.5 ± 1.1; PBS 10.1 ± 2.6 and A91 5.7 ± 1.1 (*P* = 0.001; A91 *versus* PBS; Student's *t*-test). Finally, in the case of 2 mm caudal to the epicenter, the amount of apoptotic cells was 2.1 ± 0.6 for sham animals, 12.8 ± 2.5 for PBS, and 5.1 ± 1.4 for A91-immunized rats (*P* = 0.0001; A91 *versus* PBS; Student's *t*-test).

**Figure 2:**
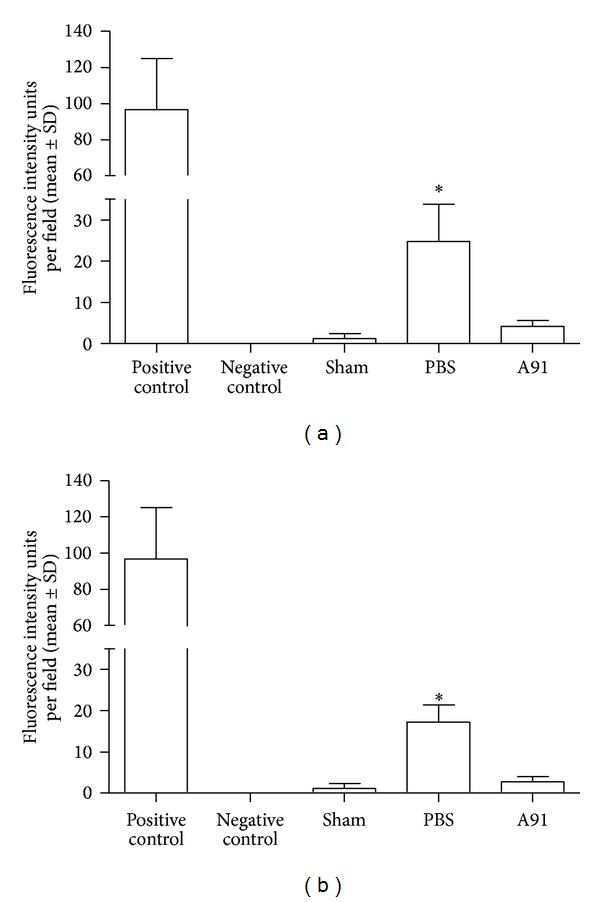
Reduction of apoptosis by A91 immunization as revealed by TUNEL assay in the injury site. Seven days after SC injury. (a) Rostral region 2 mm from injury epicenter. *Different from A91-immunized rats (*P* = 0.04; Mann-Whitney *U* test). (b) Caudal region 2 mm from injury epicenter. *Different from A91-immunized rats (*P* = 0.004; Mann-Whitney *U* test). A91 immunization induced a significant reduction in apoptosis. Bars represent the mean ± SD of 4 rats.

Figure 2 depicts the fluorescence intensity shown by sham-operated and PBS or A91-immunized rats after TUNEL analysis. In this case, we only assessed 2 mm rostral and caudal to the site of injury. As can be seen, A91 immunization reduced the apoptosis. In rostral to the epicenter case, apoptosis was significantly reduced in A91-immunized rats (4.2 ± 1.4; mean ± SD; fluorescence intensity units) as compared to PBS-immunized ones (24.8 ± 1.9; *P* = 0.04; Mann-Whitney *U* test). In the case of 2 mm caudal to the epicenter, a significant reduction of fluorescence intensity in A91-immunized animals (2.7 ± 1.2) in comparison with PBS-treated rats was also observed (17.2 ± 4.1; *P* = 0.004; Mann-Whitney *U* test).

### 3.2. Reduction in Apoptotic Cells Was Associated with a Decrease in Caspase-3 Activation

**Figure 3:**
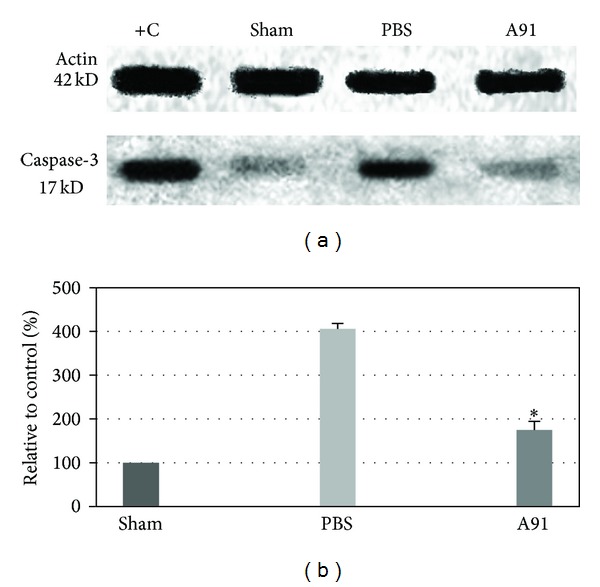
Activity of caspase-3 seven days after SC injury and PBS or A91 immunization. (a) Representative Western blots of caspase-3 expression. (b) Expression levels of caspase-3 after densitometric analysis. A91 immunization reduced the activity of caspase-3. *Different from PBS-immunized rats (*P* < 0.05; Student's *t*-test). Bars represent the mean ± SD of 6 rats. This is one of three independent Western blot assays performed in the 6 rats of each group, where we observed the same effect. Jurkat cells were used as a positive control (C+).

In order to substantiate the previous results, we analyzed the activation of caspase-3 in the SC of the other 3 groups of animals. As observed in Figure 3, A91 significantly reduced caspase-3 activation (175 ± 19.5; mean ± SD; percentage relative to sham rats). The decrease in cleaved caspase-3 of this group was more than 50% compared to PBS-immunized rats (405.93 ± 12.6; *P* < 0.05; PBS *versus* A91; Student's *t*-test).

### 3.3. A91 Immunization Also Induced a Decrease in TNF-**α** Concentrations

**Figure 4:**
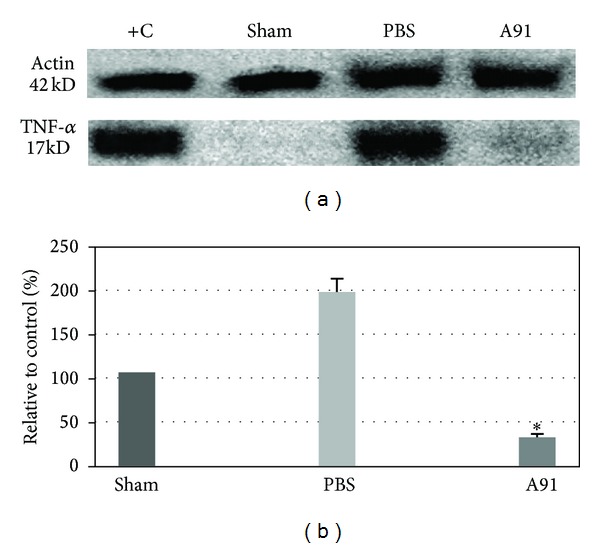
Expression of TNF-*α* seven days after SC injury and PBS or A91 immunization. (a) Representative Western blots of TNF-*α* expression. (b) Expression levels of TNF-*α* after densitometric analysis. A91 immunization reduced the expression of TNF-*α*. *Different from PBS-immunized rats (*P* < 0.001; Student's *t*-test). Bars represent the mean ± SD of 6 rats. This experiment is one of three in which we observed the same effect. L929 cells were used as a positive control (C+).

In order to elucidate another mechanism involved in the reduction of apoptosis, we determined TNF-*α* levels. This cytokine is upregulated after SC injury and is strongly related to apoptosis [24, 25]. The rationale to investigate TNF-*α* was based on our previous results where we demonstrated that A91 induces a predominant Th2 phenotype [14]. A decrease in TNF-*α* might correlate with a reduction in apoptotic cells. As expected, Figure 4 shows that animals immunized with A91 presented a significant decrease in the concentrations of TNF-*α* (30.5 ± 3.6; mean ± SD; percentage relative to sham animals) compared to the PBS control group (185.4 ± 4.9; *P* < 0.001; PBS *versus* A91; Student's *t*-test).

### 3.4. Reduction in Apoptosis Could Be Improving Motor Recovery

**Figure 5:**
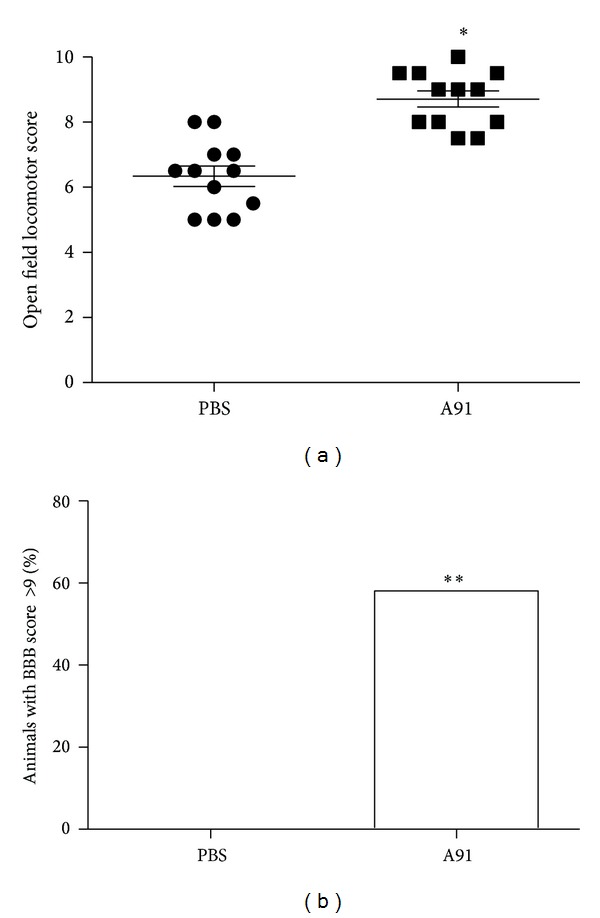
Motor recovery of rats subjected to a spinal cord contusion and immunized with A91 or PBS. Thirty days after SC injury, BBB scores showed a better improvement in rats receiving A91 immunization (a). *Different from PBS (*P* < 0.001; Student's *t*-test). Bars represent the mean ± SD of 12 rats. A significant percentage of animals attained BBB scores of 9 and 10 in A91-immunized rats (b). **Different from PBS group (*P* ≤ 0.05; Fisher exact probability test).

Finally, we evaluated the motor recovery of SC-injured animals immunized either with PBS or A91. As we have already demonstrated the positive effect of A91 immunization on motor recovery (refer to [11, 13, 14]), we only evaluated the recovery 30 days after injury. The motor recovery observed in A91-immunized rats (8.72 ± 0.2; mean ± SD) was significantly higher than the one presented by PBS-immunized animals (6.2 ± 0.3; *P* < 0.001; Student's *t*-test; Figure 5(a)). Interestingly, 58% of A91-immunized animals presented BBB scores of 9 or 10. This finding is relevant since it refers to a clinical recovery from weight support in stance to occasional weight-supported plantar steps (Figure 5(b)). None of the animals from the PBS group presented such motor recovery.

## 4. Discussion

At the moment, there is substantial evidence supporting the critical role that apoptotic cell death plays after SC injury. This process emerges as a crucial factor that contributes to the ongoing cell loss following CNS injury [26]. After SC injury, there is widespread apoptosis of neurons, oligodendroglia, and microglia [27]. The cells exhibiting the apoptotic phenotype have even been located several millimeters away from the injury epicenter. This observation indicates that apoptosis contributes to tissue loss and thus to long-term neurological dysfunction [28]. Several strategies are currently being tested in order to diminish cell death as a consequence of this phenomenon. For instance, zDEVD-fmk is a caspase 3-inhibitor that reduces secondary tissue injury and improves motor function after local administration in animals with SC injury. Similarly, z-LEHD-fmk, a caspase-9 inhibitor, has a beneficial effect after SC injury. Minocycline is perhaps the compound with the most possibilities of being tested in clinical trials. It is a tetracycline that crosses the blood-brain barrier and prevents caspase upregulation, thus preventing this apoptotic phenomenon [29].

The best strategy to protect neural tissue after injury will have to take into consideration other mechanisms of damage besides apoptosis. For instance, inflammation, lipid peroxidation, neurotoxicity, and other destructive events should also be neutralized. INDP is a new strategy that has shown to have an impact on several different destructive phenomena. A number of studies evaluating this strategy have demonstrated its positive effect on glutamate, lipid peroxidation, and nitric oxide reduction [8, 13, 30]. Furthermore, this strategy has rendered important evidence on its neurorestorative effects after SC injury [10, 31–33]. The present work demonstrates for the first time the effect of INDPs on apoptosis. There was a significant reduction of apoptotic cells at the epicenter and nearby areas in the SC of animals treated with A91 immunization. A significant reduction in caspase-3 activation was observed as well. One of the main factors involved in reducing apoptosis could be A91's effect on free radical production. It is well known that reactive oxygen or nitrogen species play a basic role in the induction of apoptosis. After SC injury, a substantial production of NO takes place [34]; this uncontrolled synthesis induces the cytotoxic effects that initiate apoptosis [35]. A91 immunization is a therapy that diminishes NO production. Its beneficial effect could be, in part, related to the downregulation of iNOS gene expression [13].

INDPs could be reducing iNOS gene expression through the action of molecules like interleukin- (IL-)10 and IL-4, which are predominantly released after INDPs [14, 36]. These molecules differentially affect the signaling cascade that activates the expression of the iNOS gene.

At the same time, NO production could also be decreased as a result of the competition for L-arginine between iNOS and arginase. The Th2prevalent phenotype developed after INDPs enriches the microenvironment with IL-4, a cytokine that in addition to inhibiting iNOS gene expression increases arginase activity by inducing its production [37]. Since L-arginine is the substrate for iNOS and arginase, a plausible mechanism of IL-4-mediated inhibition of NO synthesis could also be via depletion of L-arginine through increased arginase activity.

Another mechanism by which A91 immunization could be reducing apoptosis is through the action of BDNF. Previous studies have shown that anti-MBP T cells release neurotrophic factors [38, 39]. Furthermore, anti-A91 T lymphocytes are capable of releasing significant amounts of BDNF [14]. The antiapoptotic effects of BDNF/TrkB signaling against oxidative stress have been strongly evidenced [40]. The way that BDNF promotes an antiapoptotic effect appears to be mainly through the PI3K/Akt signaling pathway via Trk receptors. Akt phosphorylates BAD, thereby inhibiting its proapoptotic functions. Akt also directly phosphorylates and inhibits the caspase proteases including caspase-9. Finally, the PI3K/Akt pathway suppresses the expression of death genes [41].

In an effort to elucidate other possible antiapoptotic effects induced by INDPs, we evaluated the action of this therapy on the expression of TNF-*α*, a well-known proapoptotic molecule, that is, elevated after SC injury [7]. TNF-*α* can induce apoptosis in nonimmune tissues via the death domain of its cell surface receptor, TNF-R1. In this case, A91 immunization also induced a significant reduction of this molecule. The exact mechanism by which INDPs reduce TNF-*α* should be explored in future studies; however, a possible mechanism could be the effect of this strategy on the induction of a Th2 (anti-inflammatory) predominant phenotype [14]. A prevalent Th2 phenotype could inhibit the progression of an inflammatory response and thereby decrease the amount of TNF-*α* released by some inflammatory cells like macrophages.

Although in the present work TNF-*α* reduction was associated with diminished apoptosis, it is worth mentioning that the role of this molecule after SC injury is still controversial. There are several studies showing TNF-*α*'s capacity to be both, pro- and antiapoptotic [7]. This is a topic that should be elucidated in further studies.

Finally, we also demonstrated that A91 immunization induces a better motor recovery as a significant percentage of animals presented weight support in stance or occasional weight-supported plantar steps after treatment. This effect is clinically relevant and could be related to the reduction in apoptotic cells. In this regard, previous studies in our laboratory had already shown that A91 immunization induces a significant neurological improvement. In such cases, the improved outcome was associated with a reduction in lipid peroxidation and nitric oxide production, two elements that are strongly related to apoptosis [8, 13].

Apoptotic cell death is observed in both neurons and oligodendrocytes and occurs predominantly in the white matter. Apoptosis of both these cell types may contribute greatly to the neurological impairment of individuals with SC injury [7]. One of the factors contributing to the functional improvement in rats immunized with A91 could be the decrease of apoptotic cells at the site of injury.

Further studies should be directed towards defining the exact mechanisms by which INDPs reduce apoptosis after SC injury.

## 5. Conclusion

A91 immunization reduces the number of apoptotic cells after SC injury. This reduction was associated with decreased TNF-*α* release. The present study shows the beneficial effect of INDPs on apoptosis and provides more evidence on the neuroprotective mechanisms exerted by this strategy.
